# *KRAS* codon 61, 146 and *BRAF* mutations predict resistance to cetuximab plus irinotecan in *KRAS* codon 12 and 13 wild-type metastatic colorectal cancer

**DOI:** 10.1038/sj.bjc.6605177

**Published:** 2009-07-14

**Authors:** F Loupakis, A Ruzzo, C Cremolini, B Vincenzi, L Salvatore, D Santini, G Masi, I Stasi, E Canestrari, E Rulli, I Floriani, K Bencardino, N Galluccio, V Catalano, G Tonini, M Magnani, G Fontanini, F Basolo, A Falcone, F Graziano

**Affiliations:** 1 Unit of Medical Oncology 2, Azienda-Ospedaliero Universitaria Pisana, Istituto Toscano Tumori and Department of Oncology, Transplantes and New Technologies in Medicine, University of Pisa, Via Roma 67 – 56126 Pisa, Italy; 2 Section of Biochemistry and Molecular Biology “G. Fornaini”, Department of Biomolecular Sciences, University of Urbino, Via Saffi 2 – 61029 Urbino Italy; 3 Unit of Medical Oncology, University Campus Biomedico, Via Alvaro del Portillo 200 – 00128 Rome, Italy; 4 Mario Negri Institute for Pharmacological Research, Via La Masa 19 – 20156 Milan, Italy; 5 Unit of Medical Oncology, San Raffaele Scientific Institute, Via Olgettina 60 – 20132 Milan, Italy; 6 Unit of Medical Oncology, Hospital of Pesaro, Via Lombroso 1 – 61100 Pesaro, Italy; 7 Division of Pathology, Department of Surgery, University of Pisa, Istituto Toscano Tumori, Via Roma 67 – Pisa, Italy

**Keywords:** colorectal cancer, cetuximab, *KRAS*, *BRAF*

## Abstract

**Background:**

*KRAS* codons 12 and 13 mutations predict resistance to anti-EGFR monoclonal antibodies (moAbs) in metastatic colorectal cancer. Also, *BRAF* V600E mutation has been associated with resistance. Additional *KRAS* mutations are described in CRC.

**Methods:**

We investigated the role of *KRAS* codons 61 and 146 and *BRAF* V600E mutations in predicting resistance to cetuximab plus irinotecan in a cohort of *KRAS* codons 12 and 13 wild-type patients.

**Results:**

Among 87 *KRAS* codons 12 and 13 wild-type patients, *KRAS* codons 61 and 146 were mutated in 7 and 1 case, respectively. None of mutated patients responded *vs* 22 of 68 wild type (*P*=0.096). Eleven patients were not evaluable. *KRAS* mutations were associated with shorter progression-free survival (PFS, HR: 0.46, *P*=0.028). None of 13 *BRAF*-mutated patients responded *vs* 24 of 74 *BRAF* wild type (*P*=0.016). *BRAF* mutation was associated with a trend towards shorter PFS (HR: 0.59, *P*=0.073). In the subgroup of *BRAF* wild-type patients, *KRAS* codons 61/146 mutations determined a lower response rate (0 *vs* 37%, *P*=0.047) and worse PFS (HR: 0.45, *P*=0.023). Patients bearing *KRAS* or *BRAF* mutations had poorer response rate (0 *vs* 37%, *P*=0.0005) and PFS (HR: 0.51, *P*=0.006) compared with *KRAS* and *BRAF* wild-type patients.

**Conclusion:**

Assessing *KRAS* codons 61/146 and *BRAF* V600E mutations might help optimising the selection of the candidate patients to receive anti-EGFR moAbs.

RAS and RAF proteins play a key role in the control of cellular growth, proliferation and differentiation (Bos, 1989; Wickenden *et al*, 2008) *KRAS*-activating mutations reduce or abolish intrinsic GTPase activity of the protein, leading to its constitutive activation (Conlin *et al*, 2005) Similarly *BRAF* V600E mutation induces structural changes in RAF protein which increase its kinase activity (Wan *et al*, 2004) Activated RAS and RAF are responsible for the disregulation of RAS/RAF/MAPKs signalling pathway.

*KRAS* codons 12 and 13 activating mutations are widely recognised as predictors of resistance to the treatment with anti-EGFR monoclonal antibodies (moAbs) in metastatic colorectal cancer (mCRC) patients (Karapetis *et al*, 2008; Amado *et al*, 2008). Based on retrospectively collected data and *post hoc* analyses of large phase III studies, the European Medicines Agency has restricted the use of cetuximab to the treatment of patients with *KRAS* codons 12 and 13 wild-type disease (about the 60% of the overall population; EMEA, 2008) and the American Society of Clinical Oncology has similarly recommended in a recent provisional clinic opinion not to administer anti-EGFR moAbs to patients with *KRAS* codons 12 or 13 mutated tumours (Allegra *et al*, 2009). Nevertheless, in a systematic review and meta-analysis, Linardou *et al* reported a very high specificity (0.93, (0.83–0.97)) and a much lower sensitivity (0.47, (0.43–0.52)) of *KRAS* analysis in predicting resistance to anti-EGFR moAbs in mCRC, thus underlining the need for additional predictive markers to be combined with *KRAS* codons 12 and 13 evaluation, for a more accurate patients' selection (Linardou *et al*, 2008).

A recently published experience found a correlation between *BRAF* V600E-activating mutation, mutually exclusive with *KRAS* ones, and resistance to the treatment with cetuximab and panitumumab administered alone or in combination with chemotherapy (Di Nicolantonio *et al*, 2008).

On the basis of the above-mentioned evidences, around 50% of candidate patients would be excluded from receiving anti-EGFR moAbs, with a significant improvement of the treatment's cost effectiveness. However, as cetuximab or panitumumab monotherapies determine a response rate of about 10% regardless of the line of treatment and no more than 23% of patients respond to the combination of cetuximab and irinotecan (Cunningham *et al*, 2004; Van Cutsem *et al*, 2007; Jonker *et al*, 2007) it is plausible that the selection of candidate patients to receive an anti-EGFR moAb might be further slightly refined.

Additional *KRAS*-activating mutations, involving codons 61 and 146 (Edkins *et al*, 2006) occur with frequencies ranging from 1 to 4% in CRCs. These relatively rare mutations, as well as codons 12 and 13 mutations, are responsible for the oncogenic constitutive activation (Buhrman *et al*, 2007; Feig and Cooper, 1988) of RAS/RAF/MAPKs pathway and they might account for up to a 10% of resistant patients bearing *KRAS* codons 12 and 13 and *BRAF* wild-type tumours.

To optimise the selection of patients who are more likely to benefit from anti-EGFR we investigated in a cohort of patients treated with the combination of cetuximab and irinotecan and bearing *KRAS* codons 12 and 13 wild-type tumours, the association of *KRAS* codons 61 and 146 mutations and *BRAF* V600E mutation with clinical outcomes.

## Materials and methods

### Patients

Patients with irinotecan-refractory mCRC (ie, progressed during or within 3 months after treatment with an irinotecan-based regimen) were considered eligible for our study if they had an histologically confirmed diagnosis of EGFR-positive adenocarcinoma, measurable and evaluable disease according to RECIST criteria (Therasse *et al*, 2000) available paraffin-embedded samples of primary lesions and had undergone a salvage cetuximab-irinotecan treatment.

All patients' samples were screened for *KRAS* codons 12 and 13 mutations, constituting the group named ‘overall population’ and only those with wild-type disease were included in the group named ‘study population’ and further analysed for *KRAS* codons 61, 146 and *BRAF* V600E mutations.

Response was evaluated every 8 weeks by CT-scan. According to RECIST criteria, patients were categorised as responders if they achieved complete response (CR) or partial response (PR), or non-responders if they showed stable (SD) or progressive disease (PD). Progression-free survival (PFS) was defined as the time from the beginning of chemotherapy to first appearance of progression or death by any cause. Overall Survival (OS) was defined as the time from the beginning of therapy to death or last follow-up (censored observations).

Patients' characteristics and their outcomes were unknown to investigators performing genetic analyses. The study was approved by local Ethical Committees and patients provided signed informed consent to mutational analyses.

### Mutational analyses

Mutational analyses were centralised and performed at the Laboratory of Molecular Biology, Institute of Biochemistry, University of Urbino.

DNA was extracted from tissue samples using the Qiamp DNA FFPE tissue Kit (Qiagen, Hilden, Germany) according to the manufacturer's protocol.

Hotspot mutation sites were amplified by polymerase chain reaction (PCR). Primer sequences and cycling conditions are shown in Table 1. Primers design was performed by mean of PSQ Assay Design Software (Biotage, Uppsala, Sweden).

Each PCR reaction contained 50–150 ng of DNA, 0.4 *μ*M of each primer, 12.5 *μ*l of PCR Master Mix (Diatheva, Fano, Italy) and 0.625 U of HotStarTaq polymerase (Diatheva) in a total volume of 25 *μ*l. Successful and specific amplification of the region of interest was verified by visualising 5 *μ*l of the PCR product on a 2% agarose gel.

Preparation of the single-stranded DNA template for pyrosequencing analysis was performed using the PSQ Vacuum Prep Tool (Biotage) according to the manufacturer's instructions. A portion of 20 *μ*l of biotinylated PCR product was immobilised on streptavidin-coated Sepharose High-Performance beads (Amersham Biosciences, Piscataway, NJ, USA) and processed to obtain a single-stranded DNA using the PSQ 96 Sample Preparation Kit (Biotage) according to the manufacturer's instructions.

The template was incubated with 0.4 *μ*mol l^−1^ sequencing primer at 80°C for 2 min in a PSQ96 plate. The sequencing by synthesis reaction of the complementary strand was automatically performed on a PSQ 96MA instrument (Biotage) using PyroGold reagents (Biotage).

### Statistical analysis

Results of *KRAS* and *BRAF* mutational analyses were used as categorical variables (presence or absence of the mutation). The primary end-point was the correlation between *KRAS* codon 61, 146, *BRAF* V600E mutations and response to treatment in the study population. Two-tailed Fisher's exact test was used to compare proportions of responders and non-responders according to their mutational status. The PFS and OS analyses were determined according to the Kaplan–Meier method and survival curves were compared using the log-rank test. Statistical significance was set at *P*<0.05 for a bilateral test.

## Results

A total of 138 patients with mCRC, who had received cetuximab and irinotecan in four Italian Medical Oncology Units, have been screened for *KRAS* codons 12 and 13 mutations.

Clinical and pathological characteristics of the overall population and of the study population are summarised in Table 2.

Eighty-seven (63%, 52 men and 35 women) of 138 patients had *KRAS* codons 12 and 13 wild-type disease and entered the study population. Median age was 66 (range: 41–79). Of these 87, 44 (51%), 39 (44%) and 4 (5%) patients had an ECOG PS of 0, 1 and 2, respectively.

All patients received cetuximab plus irinotecan according to the schedule commonly used in clinical practice: cetuximab 250 mg/sqm i.v., day 1 weekly (loading dose: 400 mg/sqm i.v., day 1 in the first cycle) or 500 mg/sqm i.v., day 1 every 2 weeks and irinotecan 180 mg/mq i.v., day 1 every 2 weeks.

In the study population, according to RECIST criteria, 1 CR and 23 PRs were reported, for an overall response rate (RR) of 28%. Of the patients, 35 (40%) achieved SD and 28 (32%) experienced PD. At the time of the analysis, 81 (93%) patients underwent disease progression and 63 (72%) died. Median PFS and median OS were 4.1 and 9.7 months, respectively.

### Mutational status of *KRAS* codons 61 and 146

**Figure 1:**
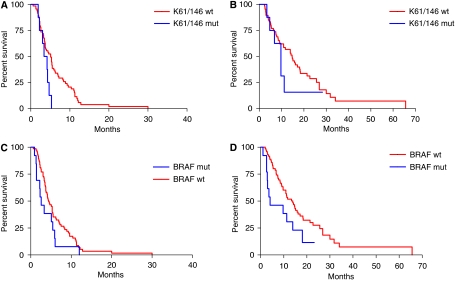
(**A**) Progression-free survival and (**B**) overall survival curves according to *KRAS* codons 61 and 146 mutational status in study population. (**C**) Progression-free survival and (**D**) overall survival curves according to *BRAF* codon 600 mutational status in study population.

The mutational analyses of codons 61 and 146 were successfully performed in 76 out of 87 cases. *KRAS* was mutated in codon 61 or 146 in 7 (8%) and 1 (1%) cases, respectively. None of the 8 patients bearing *KRAS* 61 or 146 mutated disease responded to the treatment, whereas 22 (32%) of 68 patients with wild-type disease achieved response (*P*=0.096; Table 3B). *KRAS* 61 and 146 mutations were associated with significantly shorter PFS (median PFS: 3.8 *vs* 5.1 months in *KRAS* 61 and 146 wild type; HR: 0.46 (0.11–0.88), *P*=0.028; Figure 1A), whereas no significant differences were detected in OS (median OS: 9.7 *vs* 14.7 months in *KRAS* 61 and 146 wild-type; HR: 0.69 (0.24–1.75), *P*=0.390; Figure 1B).

### Mutational status of *BRAF* codon 600

Among the 87 patients of the study population, *BRAF* was mutated in 13 cases (15%). *KRAS* codons 61, 146 and *BRAF* V600E mutations were mutually exclusive. None of the patients bearing *BRAF* mutation responded to the treatment, in comparison with 24(32%) of 74 patients with *BRAF* wild-type disease (*P*=0.016; Table 3C). *BRAF* mutation was associated with a trend towards shorter PFS (median PFS: 2.6 *vs* 4.4 months in *BRAF* wild-type; HR: 0.59 (0.24–1.07), *P*=0.073; Figure 1C) and with significantly shorter OS (median OS: 4.1 *vs* 13.9 months in *BRAF* wild-type; HR 0.51 (0.18–0.95), *P*=0.037; Figure 1D).

### Combined analysis of *KRAS* codons 61 and 146 and *BRAF* codon 600 mutational status

**Figure 2:**
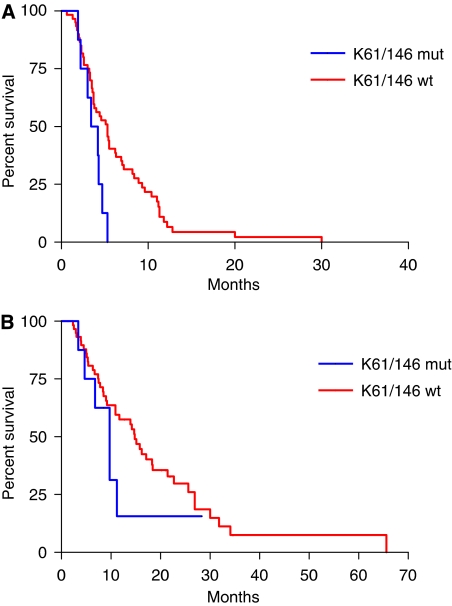
(**A**) Progression-free survival and (**B**) overall survival curves according to *KRAS* codons 61 and 146 mutational status in *BRAF* wild-type subgroup.

Among 74 patients with *BRAF* wild-type disease, *KRAS* codons 61 and 146 mutational status was successfully assessed in 68 cases. Eight (11%) tumours were *KRAS* 61 or 146 mutated. None of patients with *KRAS* 61 or 146 mutated tumours responded, in comparison with 22 (37%) of 60 *KRAS* 61 and 146 wild-type patients (*P*=0.047; Table 4A). Also in *BRAF* wild-type subpopulation, *KRAS* codons 61 and 146 mutations were associated with shorter PFS (median PFS: 3.8 *vs* 5.3 months in *KRAS* 61 and 146 wild-type; HR: 0.45 (0.10–0.85), *P*=0.023; Figure 2A), whereas no significant differences were reported in terms of OS (median OS: 9.7 *vs* 14.8 months in *KRAS* 61 and 146 wild-type; HR: 0.32 (0.21–1.66), *P*=0.320; Figure 2B).

**Figure 3:**
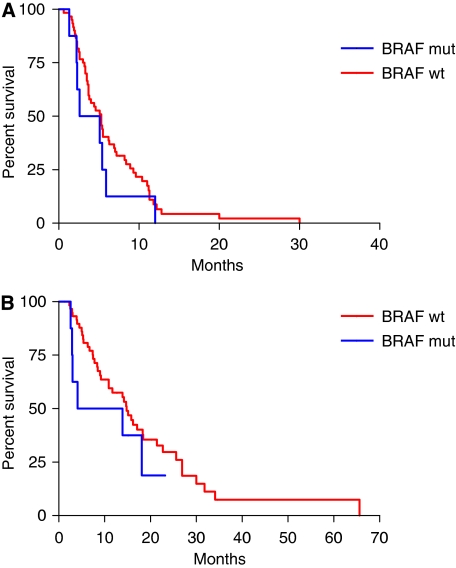
(**A**) Progression-free survival and (**B**) overall survival curves according to *BRAF* codon 600 mutational status in *KRAS* codon 61 and 146 wild-type subgroup.

In the subgroup of 68 patients with *KRAS* 61 and 146 wild-type tumours, *BRAF* was mutated in 8 cases. None of *BRAF*-mutated patients obtained a response, whereas 22 (37%) out of 60 patients with *BRAF* wild-type disease responded (*P*=0.047; Table 4B). *BRAF* mutation was associated with a trend towards shorter PFS (median PFS: 3.9 *vs* 5.3 months in *BRAF* wild-type; HR: 0.69 (0.26–1.52), *P*=0.306; Figure 3A) and OS (median OS: 9.0 *vs* 14.8 months in *BRAF* wild-type; HR: 0.63 (0.20–1.60), *P*=0.279; Figure 3B).

**Figure 4:**
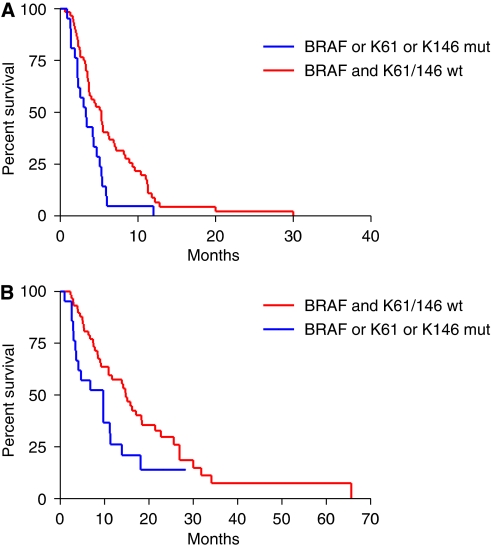
(**A**) Progression–free survival and (**B**) overall survival curves of patients with *KRAS* codon 61 or 146 or *BRAF*-mutated disease compared to those of patients with wild-type disease.

Among 87 patients of the study population, none of 21 patients with tumours presenting whatever mutation responded to the treatment, in comparison with 22 (37%) of 60 patients with *KRAS* 61 and 146 and *BRAF* wild-type disease (*P*=0.0005; Table 5). Wild-type patients reported significantly longer PFS (median PFS: 5.3 *vs* 3.3 months; HR: 0.51 (0.22–0.77), *P*=0.006; Figure 4A) and OS (median OS: 14.8 *vs* 9.7 months; HR: 0.54 (0.24–0.92), *P*=0.027; Figure 4B).

## Discussion

The molecular test for *KRAS* mutations has been introduced in the routine clinical practice of the oncologists facing mCRC (Allegra *et al*, 2009). Since the first report of *KRAS* predictive value for resistance to cetuximab (Lièvre *et al*, 2006), investigators looked at most of the frequent mutations (ie, those affecting codons 12 and 13). Therefore, also the most reliable demonstrations of *KRAS* mutations as determinants of resistance to anti-EGFR moAbs, that is, those deriving from *post hoc* analyses of randomised studies (Karapetis *et al*, 2008; Amado *et al*, 2008; Van Cutsem *et al*, 2009), investigated only codons 12 and 13 genetic variants. Even if quite specific for non-responsiveness, *KRAS* codon 12 and 13 analysis suffers from low sensitivity. In fact around 35% of wild-type patients experience rapid disease progression.

This background led to the search for alternative predictive factors, such as EGFR ligands expression (Khambata-Ford *et al*, 2007) or polymorphisms (Garm Spindler *et al*, 2009), alterations in other EGFR signalling pathways (ie, PTEN/PI3K/AKT; Loupakis *et al*, 2009) or in downstream effectors of *KRAS* (ie, *BRAF*). In particular, Di Nicolantonio *et al* (2008) recently found that none of 11 *BRAF*-mutated patients, among 79 *KRAS* codon 12 and 13 wild type, responded to anti-EGFR moAbs. *BRAF* mutation also predicted for an unfavourable outcome in terms of both PFS and OS. From a methodological standpoint mutational analyses are quite more appealing with respect to other techniques such as immunohistochemistry or gene expression profiling. In fact, the determination of mutational status is easily reproducible, qualitative, less expensive and does not require fresh tumour tissue sampling. Taking into account these considerations and the growing knowledge on minor oncogenic *KRAS* mutations in codons 61 and 146 (mutually exclusive with those in codons 12 and 13), we conducted this retrospective study to verify whether the combined analyses of such rare *KRAS* mutations and *BRAF* codon 600 variants are related to resistance to cetuximab plus irinotecan. In the present analysis, 13, 7 and 1 patient among 87 patients with *KRAS* codon 12 and 13 wild-type disease had tumour bearing *BRAF* V600E, *KRAS* codon 61 and *KRAS* codon 146 mutation, respectively. None of the *KRAS* codon 12 or 13 wild-type patients bearing an alteration on *KRAS* codon 61, 146 or *BRAF* codon 600 responded to treatment. Moreover patients with mutated tumours had a significantly worse outcome both in terms of PFS and OS. Such data indicate that, even if much more rare than codon 12 or 13 mutations, codon 61 and 146 as well as *BRAF* mutations also seem to predict resistance to cetuximab. As these rare *KRAS* mutations and *BRAF* mutations are mutually exclusive with the others, it seems reasonable to test for their presence only in patients with *KRAS* codon 12 and 13 wild-type tumours. Considering our data, it seems that among those subjects that are expected not to have codon 12 or 13 mutations (around 60% of mCRC patients), testing for *BRAF* and rare *KRAS* mutations would exclude around 25% of patients, with obvious saving of economic resources and sparing unnecessary toxicities.

The quest for the most sensible and specific tools for selecting patients who are more likely to benefit from anti-EGFR inhibitors is an issue acquiring a great relevance for two main reasons. On one hand, in addition to the present indication for the use of anti-EGFR moAbs, the results from a recent phase III randomised trial (Van Cutsem *et al*, 2009) led to the approval of cetuximab for *KRAS* codon 12 and 13 wild-type patients also in the first-line setting. On the other hand, it has been proven that in such setting the choice of using an anti-EGFR moAb precludes the possibility of coadministering the antiangiogenic antibody bevacizumab, even in *KRAS* codon 12 and 13 wild-type patients (Tol *et al*, 2009; Hecht *et al*, 2009). Moreover, it should be noted that the choice of the best upfront treatment for each patient is not only to offer the best palliation, but it may even influence the possibility for cure (Adam *et al*, 2009). In this regard it should be considered that there are different ongoing randomised studies evaluating the impact of moAbs in the adjuvant setting, where the clinical impact of reliable predictors of outcome will be even greater.

In conclusion, our results suggest that *KRAS* testing power for predicting resistance to anti-EGFR might be improved by including codon 61 and 146 mutational analysis. This finding may have rapid and important implications for routine clinical practice. Moreover, this study confirms the recent finding that indicates *BRAF* V600E mutation as a promising additional marker for resistance. Such preliminary and retrospective results should be verified on samples from patients enrolled in randomised studies of anti-EGFR moAbs *vs* best supportive care.

## Figures and Tables

**Table 1:** Primers and polimerase chain reaction (PCR) conditions for mutational analyses

**Mutations**	**Primers**	**PCR conditions**
*KRAS* codon 12–13	F: 5′-GGCCTGCTGAAAATGACTGAA R: 5′-[Btn]-TTCGTCCACAAAATGATTCTGA Seq: 5′-TATAAACTTGTGGTAGTTGG	10′ at 95°C, 40 cycles of 15′′ at 95°C, 30′′ at 64°C, 40′′ at 72°C and 5′ at 72°C
*KRAS* codon 61	F: 5′-CAGACTGTGTTCTCCCTTCTCA R: 5′-[Btn]CTCATGTACTGGTCCCTCATTG Seq: 5′-ATATTCTCGACACAGCAG	10′ at 95°C, 40 cycles of 15′′ at 95°C, 30′′ at 66°C, 30′′ at 72°C and 5′ at 72°C
*KRAS* codon 146	F: 5′-TGGACAGGTTTTGAAAGATATTTG R: 5′-[Btn]-ATTAAGAAGCAATGCCCTCTCAAG Seq: 5′-AATTCCTTTTATTGAAACAT	10′ at 95°C, 40 cycles of 15′′ at 95°C, 30′′ at 64°C, 30′′ at 72°C and 5′ at 72°C
*BRAF* codon 600	F: 5′-ATGCTTGCTCTGATAGGAA R: 5′-[Btn]-GCATCTCAGGGCCAAA Seq:5′-GGTGATTTTGGTCTAGCTAC	10′ at 95°C, 40 cycles of 15′′ at 95°C, 30′′ at 54°C, 40′′ at 72°C and 5′ at 72°C

Abbrevations: Btn=biotynilated; Seq=sequencing primer.

**Table 2:** Patients' characteristics

	**Overall population (*N*=138)**	**Study population (*N*=87)**
	***n* (%)**	***n* (%)**
*Age*		
Median (range)	61 (42–77)	66 (41–79)
		
*Sex*		
Male	76 (55)	52 (60)
Female	62 (45)	35 (40)
		
*ECOG PS*		
0	70 (51)	44 (51)
1	61 (44)	39 (44)
2	7 (5)	4 (5)
		
*Number of metastatic sites*		
1	33 (24)	21 (24)
2	63 (46)	41 (47)
⩾2	42 (30)	25 (29)
		
*Sites of metastases*		
Liver	110 (80)	63 (72)
Lung	72 (52)	45 (52)
Lymph nodes	35 (25)	24 (27)
Peritoneum/pelvis	31 (22)	20 (23)
Other	46 (33)	29 (33)
		
*Skin toxicity*		
G_0_—G_1_	84 (61)	44 (51)
⩾G_2_	54 (39)	43 (49)

**Table 3:**
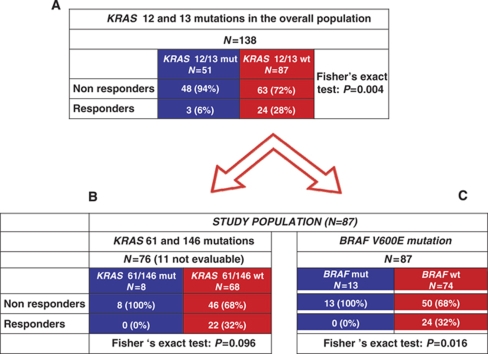
Study flow chart and correlations with response to treatment: (**A**) *KRAS* codons 12 and 13 analysis in the overall population; (B) *KRAS* 61 and 146 mutations and (**B**) *BRAF* V600E mutation in study population (*KRAS* codons 12 and 13 subgroup)

**Table 4:**
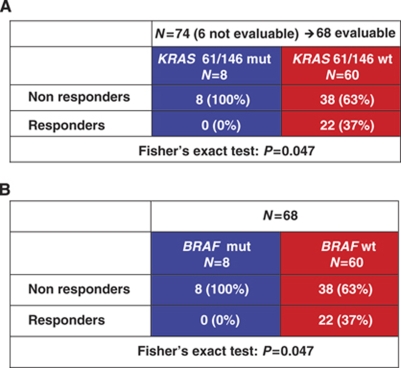
(**A**) *KRAS* 61 and 146 mutations in *BRAF* wild-type subgroup and (**B**) *BRAF* V600E mutation in *KRAS* 61 and 146 wild-type subgroup: correlation with response

**Table 5:**
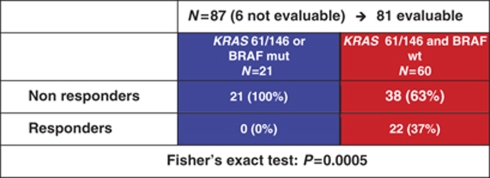
Combined analysis of *KRAS* 61/146 and *BRAF* V600E mutation in study population: correlation with response
